# Transcriptomic and free monoterpene analyses of aroma reveal that isopentenyl diphosphate isomerase inhibits monoterpene biosynthesis in grape (*Vitis vinifera* L.)

**DOI:** 10.1186/s12870-024-05306-y

**Published:** 2024-06-25

**Authors:** Tianchi Chen, Tao Xu, Jinnan Wang, Tianye Zhang, Jin Yang, Lixiao Feng, Tiefeng Song, Jian Yang, Yueyan Wu

**Affiliations:** 1 College of Biological and Environmental Sciences, Zhejiang Wanli University, Ningbo, 315100 China; 2 College of Life Sciences, Zhejiang University, Hangzhou, 310058 China; 3 State Key Laboratory for Quality and Safety of Agro-Products, Institute of Plant Virology, Ningbo University, Ningbo, 315211 China

**Keywords:** Grape, GC-MS, RNA-Seq, Monoterpene biosynthesis

## Abstract

**Background:**

Monoterpenes are among the most important volatile aromatic compounds contributing to the flavor and aroma of grapes and wine. However, the molecular basis of monoterpene biosynthesis has not yet been fully elucidated.

**Results:**

In our study, transcriptomics and gas chromatography-mass spectrometry (GC-MS) were used to mine candidate genes and transcription factors involved in monoterpene biosynthesis between high-monoterpene and zero-monoterpene table grape cultivars. We found that monoterpene biosynthesis was positively correlated by the expression of five genes encoding 1-deoxy-D-xylulose-5-phosphate synthase (*VvDXS*s), one encoding 4-hydroxy-3-methylbut-2-enyl diphosphate reductase (*VvHDR*), three hydroxy-3-methylglutaryl-CoA synthases (*VvHMGS*s) and one mevalonate kinase (*VvMVK*), whereas the expression of one isopentenyl diphosphate isomerase (*VvIDI*) and one 3-hydroxy-3-methylglutaryl-CoA reductase (*VvHMGR*) negatively correlated monoterpene biosynthesis. Of these genes, *VvIDI* was selected to validate its function in monoterpene accumulation through a transient overexpression experiment, and was shown to inhibit the biosynthesis of grape linalool and α-terpineol. Meanwhile, we found that a 64-amino acid extension sequence at the N-terminus can guide the VvIDI protein to target the chloroplast.

**Conclusions:**

The findings of this study should help to guide future functional analysis of key genes as well as mining the potential regulatory mechanism of monoterpene biosynthesis in grapes and grape products.

**Supplementary Information:**

The online version contains supplementary material available at 10.1186/s12870-024-05306-y.

## Background

Grape (*Vitis vinifera* L.), a perennial and deciduous woody vine, is widely cultivated around the world [1, 2]. Due to its delicate flavor and rich nutritional value, grape has become one of the most popular and important fruits globally in the diet of people [3]. Flavor and aroma are important characteristics that directly affect grape quality and acceptability to consumers [4]. Among the various classes of grape volatile aroma compounds, monoterpenes, including linalool, geraniol and nerol, have been widely recognized to be responsible for the characteristic varietal flavors in Muscat-type grape varieties [5]. Grape monoterpenes are often described as presenting floral and/or citrus type aromas and are the most abundant forms of terpenes found in Muscat-type table grapes [6].

Monoterpene compounds produced by plants vary widely with respect to their structure and functionalities and are synthesized from their isoprenoid precursors, isopentenyl diphosphate (IPP) and dimethylallyl pyrophosphate (DMAPP), formed via the plastidic 2-C-methyl-D-erythritol 4-phosphate (MEP) pathway and the cytoplasmic mevalonate (MVA) pathway [7]. In the MVA pathway, IPP is derived from acetyl-coenzyme A (Ac-CoA) through a series of enzymatic reactions, which involves enzymes such as 3-hydroxy-3-methylglutaryl-CoA synthase (HMGS), 3-hydroxy-3-methylglutaryl-CoA reductase (HMGR) and mevalonate kinase (MVK). In the MEP pathway, IPP and DMAPP are produced enzymatically from pyruvic acid and 3-phosphate glyceraldehyde (G3P) by several steps, starting with the 1-deoxy-D-xylulose 5-phosphate synthase (DXS), 1-deoxy-D-xylulose 5-phosphate reductoisomerase (DXR), and ending with 4-hydroxy-3-methylbut-2-enyl-diphosphate reductase (HDR). The balance between IPP and DMAPP is controlled by isopentenyl diphosphate isomerase (IDI), which reversibly converts IPP to DMAPP and influences downstream reactions [8]. Subsequently, the reaction of 1 × IPP and 1 × DMAPP to form geranyl-PP (GPP) is catalyzed by geranyl diphosphate synthase (GPPS). Finally, monoterpenes are synthesized from GPP via a series of terpene synthases (TPSs) and are released gradually from the fruit in the free state. However, the detailed molecular mechanism controlling the levels of volatile monoterpenes still needs to be elucidated.

‘Shine Muscat’ (*V. vinifera* L. × *V. labrusca* Baily), a seedless green grape variety, was formed by the hybridization of ‘Akitsu-cho 21’ and ‘Muscat of Alexandria’ in the late 1980s and was then commercialized in the early 2000s [9]. Its characteristic Muscat-type flavor was largely determined by the concentration of monoterpenes in the fruit flesh [10]. ‘Heibaladuo’ is a hybrid cultivar from a cross between ‘Yoneyama 3’ and ‘Hongbaladuo’ and has gained popularity due to its disease resistance and high yields. The monoterpene aroma profile shows differences among different cultivars. Thus, ‘Shine Muscat’ (a Muscat cultivar) and ‘Heibaladuo’ (a non-Muscat cultivar) table grapes were selected as experimental materials with which to identify the candidate genes that correlate and regulate monoterpene biosynthesis, using transcriptomics and GC-MS. Additionally, the function of *VvIDI* in monoterpene biosynthesis was validated. Our goal was that the results of this study would be able to provide insights into the regulatory mechanism by which monoterpene synthesis is controlled and ultimately would allow researchers to manipulate candidate genes to improve grape aroma.

## Results

### Determination of table grape maturity

**Fig. 1:**
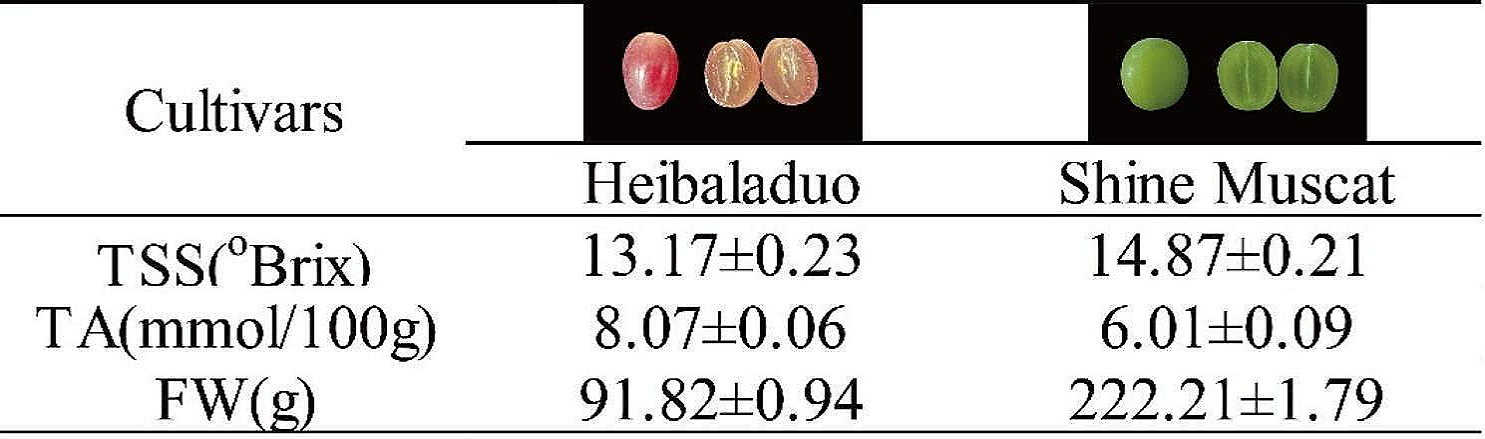
The visual appearance, total acidity (TA, mmol/100 g), total soluble solids (TSS, °Brix) and the total fresh weight (FW, g) of ‘Heibaladuo’ and ‘Shine Muscat’ table grapes

To estimate the maturity of ‘Heibaladuo’ and ‘Shine Muscat’ table grapes, their visual appearances, total soluble solids concentration (TSS), total acidity (TA) and the total fresh weight (FW) of 20 berries are presented in Fig. 1. The TSS values of the grape berries of both cultivars were greater than 12°Brix, and their TA values were all lower than 10 mmol/100 g. The TSS and FW values of ‘Shine Muscat’ were greater than in ‘Heibaladuo’. The value of each indicator indicated that the grape berries of the two cultivars had been harvested at their appropriate maturity times, so could be used for comparative (of the two cultivars) studies by GC-MS and RNA-Seq analyses.

### Identification and characterization of the volatile compounds

**Fig. 2:**
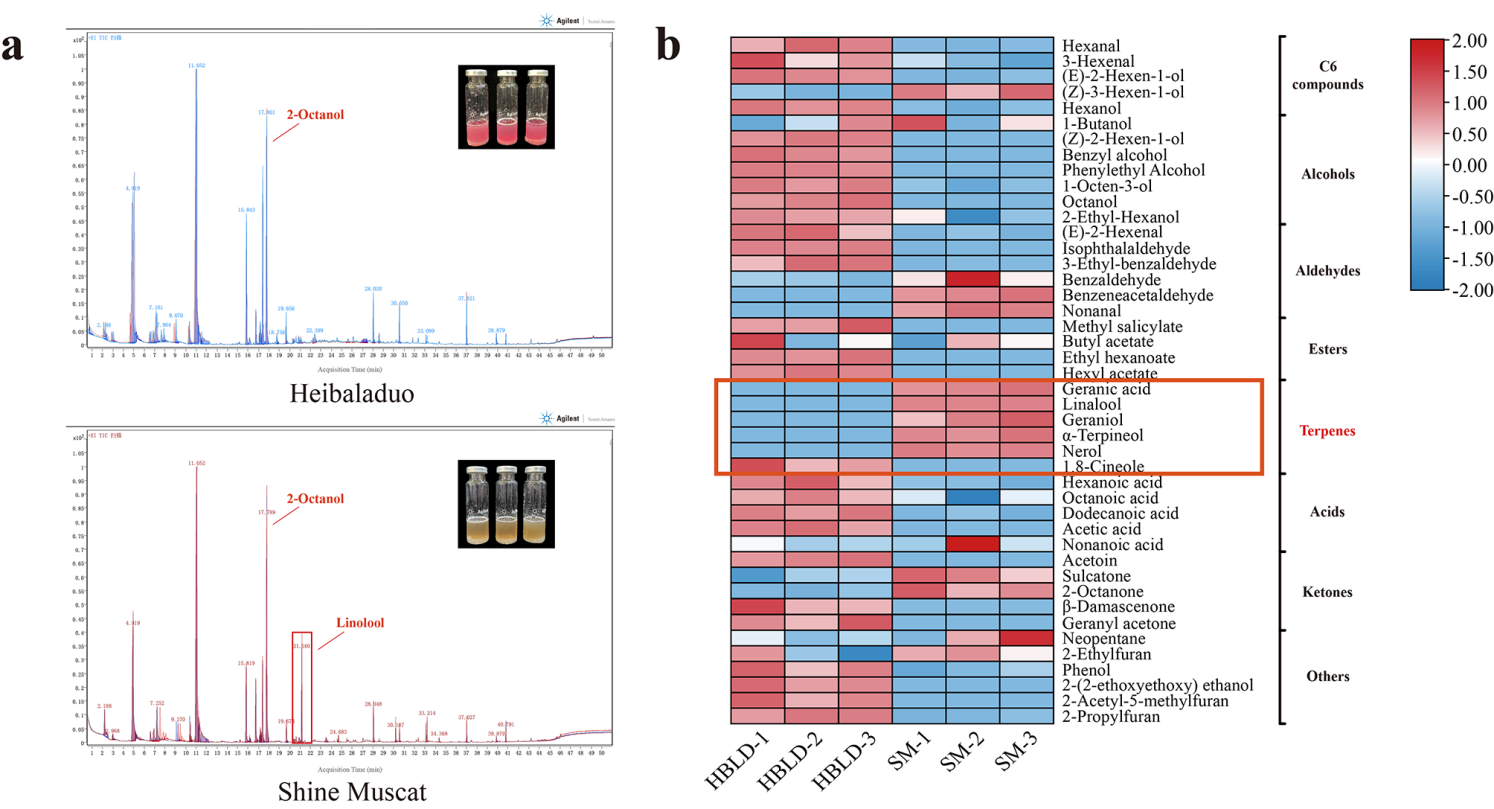
GC-MS analysis of aroma volatiles from berries of ‘Heibaladuo’ and ‘Shine Muscat’ table grapes. (**a**) Total ion chromatogram (TIC) of volatile compounds determined in the pulp juice of the two cultivars. (**b**) Heat map presenting the different volatile compounds between the two cultivars of table grape. Columns and rows represent the samples and volatile compounds, respectively. HBLD-1, HBLD-2 and HBLD-3 refer to the three replicates of ‘Heibaladuo’ and SM-1, SM-2 and SM-3 refer to the three replicates of ‘Shine Muscat’. The color from red to blue indicates high to low concentration of each volatile compound

To determine the identity and concentration of different volatile compounds in a comparison between ‘Heibaladuo’ and ‘Shine Muscat’ table grapes, the concentration of each volatile compound in the vial headspace above the grape pulp extract was obtained using GC-MS. The total ion chromatograms (TICs) of the two cultivars of table grape, with three replicates for each cultivar, are shown in Fig. 2a. The shape of each peak was sharp and steep, and the TIC curves and acquisition time of the three replicates agreed and coincided well among replicates, indicating that the GC-MS data were accurate and reliable. Forty-four free volatile aroma compounds, including five C_6_ compounds, seven alcohols, six aldehydes, four esters, six terpenes, five acids and five ketones, were identified based on the retention times (Fig. 2b). The molecular formula, CAS number and reference information of these compounds are listed in Table S1. We observed that there were obvious differences in the profiles of volatile aroma components between the grapes of the two cultivars, especially with respect to monoterpene compounds. The concentrations of alcohols, esters, acids and other volatile aroma compositions were significantly higher in ‘Heibaladuo’ table grapes. Various monoterpenes, including linalool (acquisition time 21.17 min), geraniol and α-terpineol, were accumulated to a higher concentration in ‘Shine Muscat’ table grape, whereas these monoterpene compounds were not detected in ‘Heibaladuo’ table grapes. We speculated that this difference was caused by expression differences in genes of the monoterpene biosynthesis pathway between the two cultivars.

### Genes differently expressed between the two cultivars of table grape

**Fig. 3:**
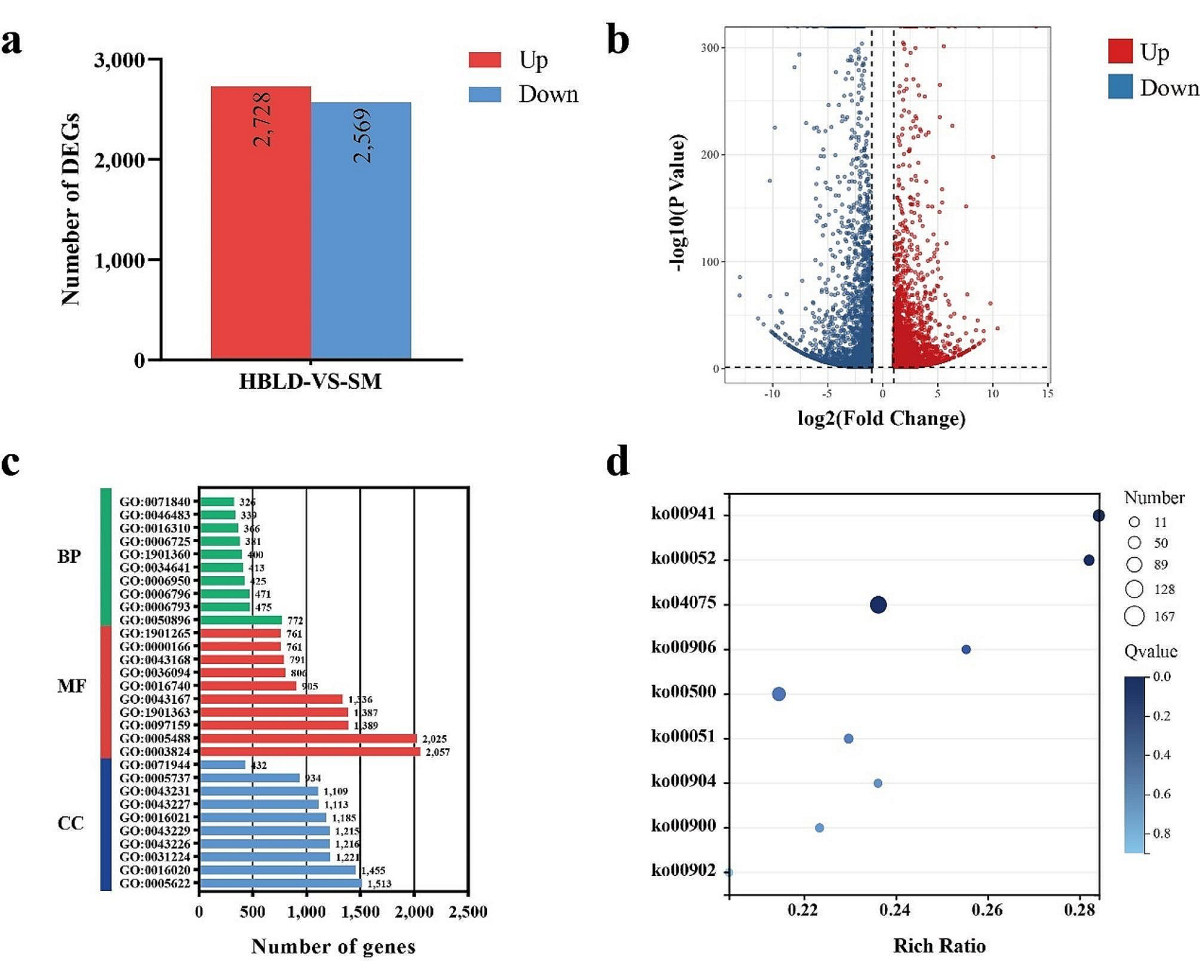
Analysis of DEGs between ‘Heibaladuo’ (HBLD) and ‘Shine Muscat’ (SM) table grapes. (**a**) Number of up- and down-regulated DEGs in HBLD vs. SM. (**b**) Volcano map of up-regulated (red plot) and down-regulated (blue plot) DEGs in HBLD vs. SM. (**c**) GO classifications in Biological Process (BP), Molecular Function (MF), and Cellular Component (CC) categories. (**d**) KEGG pathway annotation and enrichment of DEGs

To explore the differences in gene expression in berries with different monoterpene concentrations, we performed transcriptome sequencing on mature berries of the table grape varieties ‘Heibaladuo’ (HBLD) (in which monoterpenes were not detectable: “zero-monoterpene”) and ‘Shine Muscat’ (SM) (in which high monoterpene concentration were detected: “high-monoterpene”), with three biological replicates for each cultivar. A total of 5,297 differentially expressed genes (DEGs) were identified between the two cultivars. Compared with ‘Heibaladuo’, ‘Shine Muscat’ contained 2,728 significantly up-regulated DEGs and 2,569 significantly down-regulated DEGs (Fig. 3a and b). Gene Ontology (GO) enrichment analysis showed that the 5,275 DEGs were divided into 164 functional terms (Table S2). More specifically, 4,368 DEGs (a gene may be involved in multiple subcategories) were assigned to the top 10 subcategories in the “Biological Process” category, 12,218 DEGs were assigned to the top 10 subcategories in the “Molecular Function” category, and 11,393 DEGs were assigned to the top 10 subcategories in the “Cellular Component” category (Fig. 3c). Within the “Biological Process” category, the subcategories with the most DEGs were “response to stimulus” (GO:0050896), “phosphorus metabolic process” (GO:0006793) and “phosphate-containing compound metabolic process” (GO:0006796). Within the “Molecular Function” category, the subcategories with the most DEGs were “catalytic activity” (GO:0003824), “binding” (GO:0005488) and “organic cyclic compound binding” (GO:0097159). In the “Cellular Component” category, the subcategories with the most DEGs were “intracellular anatomical structure” (GO:0005622) and “membrane” (GO:0016020), followed by “intrinsic component of membrane” (GO:0031224).

KEGG enrichment analysis showed that a total of 2,333 DEGs were enriched in 134 biosynthetic pathways (Table S3), which were “metabolism” (103, 76.9%), “genetic information processing” (21, 15.7%), “cellular processes” (4, 3.0%), “environmental information processing” (4, 3.0%) and “organismal systems” (2, 1.5%). We observed that the DEGs were abundantly enriched in glycan metabolism, including “Starch and sucrose metabolism” (ko00500), “Galactose metabolism” (ko00052) and “Fructose and mannose metabolism” (ko00051) (Fig. 3d). Meanwhile, there was a large number of DEGs enriched with respect to terpene metabolism, including “Terpenoid backbone biosynthesis” (ko00900), “Carotenoid biosynthesis” (ko00906), “Diterpenoid biosynthesis” (ko00904) and “Monoterpenoid biosynthesis” (ko00902). In addition, a large number of DEGs was enriched with respect to “Plant hormone signal transduction” (ko04075) and “Flavonoid biosynthesis” (ko00941).

### Transcripts associated with the monoterpene biosynthesis pathway

**Fig. 4:**
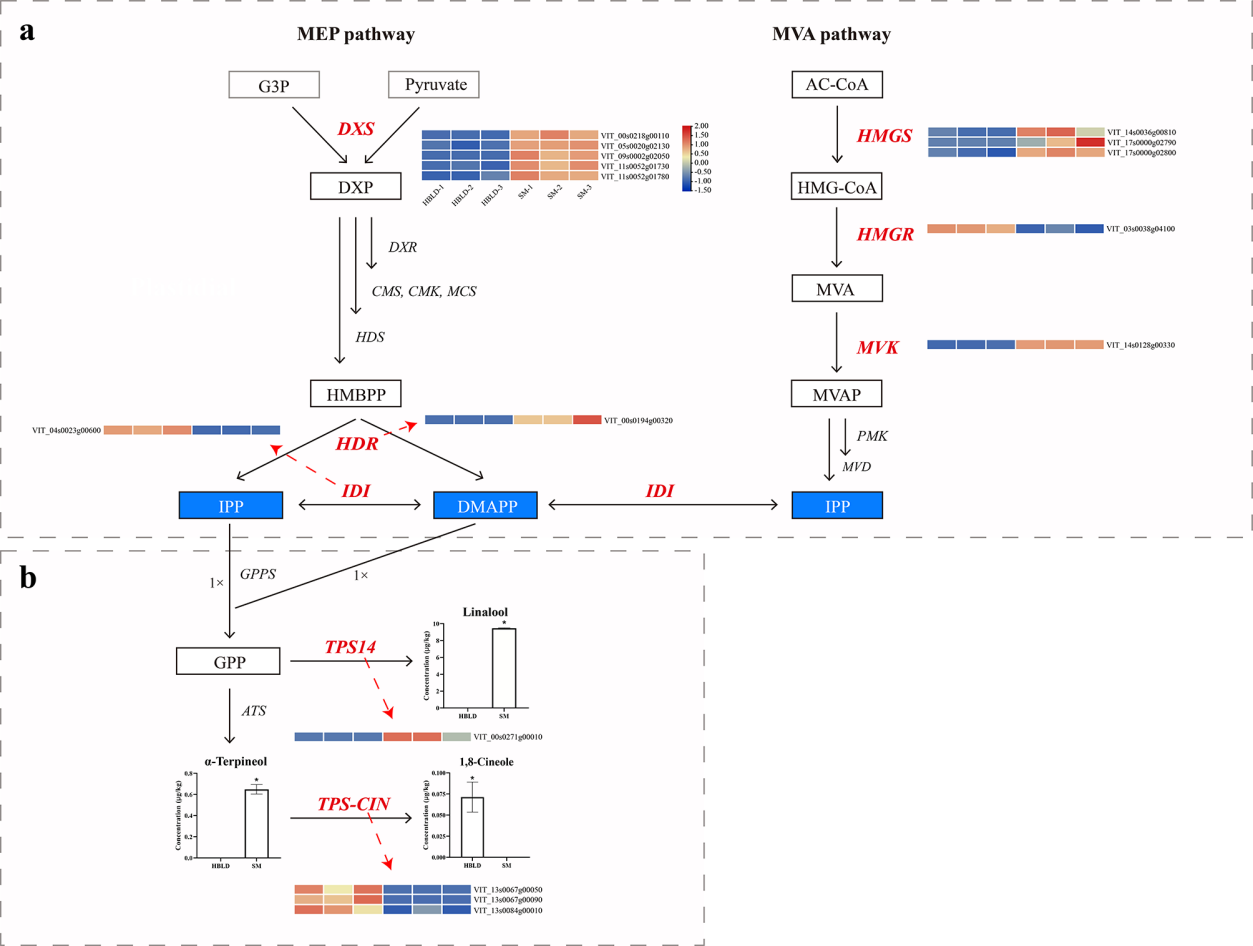
DEGs in monoterpene biosynthesis pathways. (**a**) DEGs in terpenoid backbone biosynthesis pathways. DEGs are annotated in red. Heat map presents the gene expression levels of the DEGs. The colors of the boxes represent the intensity of the gene expression FPKM value; red and blue colors reflect high and low gene expression levels, respectively. (**b**) DEGs in monoterpenoid biosynthesis pathways. Error bars represent the standard deviation, SD (*n* = 3), and an asterisk (*) above the bars represents a significant difference (*p* < 0.05)

From the RNA-Seq data, a total of 32 genes involved in the “Terpenoid backbone biosynthesis” and “Monoterpenoid biosynthesis” KEGG pathways were significantly enriched. The genes in these pathways and their transcriptional levels are presented in Fig. 4 and Table S4. Specifically, the genes encoding five 1-deoxy-D-xylulose-5-phosphate synthases (*VvDXS*s) showed higher expression in the high-monoterpene cultivar ‘Shine Muscat’ table grape than in the zero-monoterpene cultivar ‘Heibaladuo’ table grape (Fig. 4a). Of the other terpenoid backbone biosynthesis pathway genes, genes encoding three hydroxy-3-methylglutaryl-CoA synthases (*VvHMGS*s), one 4-hydroxy-3-methylbut-2-enyl diphosphate reductase (*VvHDR*) and one mevalonate kinase (*VvMVK*) also showed significantly higher expression in the ‘Shine Muscat’ table grape cultivar. Genes encoding one isopentenyl diphosphate isomerase (*VvIDI*) and one 3-hydroxy-3-methylglutaryl-CoA reductase (*VvHMGR*) were expressed at a higher level in ‘Heibaladuo’ table grapes than in ‘Shine Muscat’ table grapes. We found that monoterpenes accumulated to a high concentration in ‘Shine Muscat’ table grapes, with the predominant monoterpene in ‘Shine Muscat’, linalool, not being detected in ‘Heibaladuo’ table grapes. These findings suggest that monoterpene accumulation may be positively correlated by expression of the five *VvDXS*s, one *VvHDR*, three *VvHMGS*s and one *VvMVK*, and negatively correlated by expression of one *VvIDI* and one *VvHMGR*. In the monoterpenoid biosynthesis pathway, the expression level of the gene encoding (3 S)-linalool synthase (*VvTPS14*) was significantly higher in ‘Shine Muscat’ than in ‘Heibaladuo’ table grapes (Fig. 4b), suggesting that *VvTPS14* is a gene directly controlling the accumulation of linalool, with its high expression being able to promote linalool synthesis in ‘Shine Muscat’. In addition, genes encoding three 1,8-cineole synthases (*VvTPS-CIN*s) were more highly expressed in ‘Heibaladuo’ than in ‘Shine Muscat’ table grapes, which suggest that most of the α-terpineol was catalyzed to react further downstream by the high expression of *VvTPS-CIN*s in ‘Heibaladuo’, which caused significant differences in α-terpineol and 1,8-cineole concentrations between the two cultivars.

### Reverse transcription-quantitative PCR (RT-qPCR) validation of candidate DEGs

**Fig. 5:**
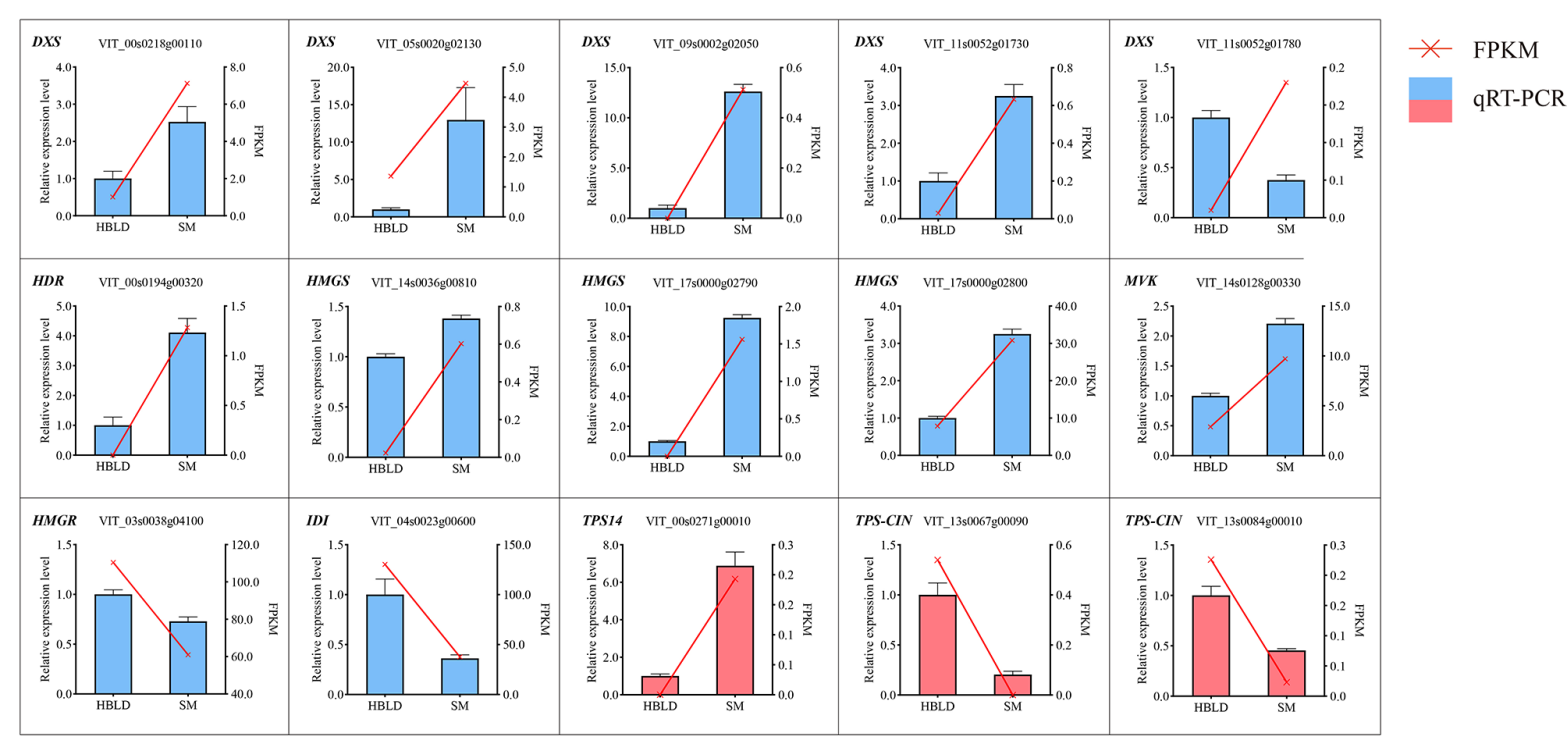
RT-qPCR validation of candidate DEGs. The blue and red columns represent the key DEGs belonging to terpenoid backbone and monoterpenoid biosynthesis pathway genes, respectively. Error bars represent the SD (*n* = 3)

To validate the reliability of the transcriptome data, a total of 15 candidate DEGs, including 12 terpenoid backbone pathway genes (five *VvDXS*s, one *VvHDR*, three *VvHMGS*s, one *VvMVK*, one *VvHMGR*, and one *VvIDI*) and three monoterpenoid biosynthesis pathway genes (one *VvTPS14* and two *VvTPS-CIN*s) were selected for RT-qPCR (Fig. 5). The results showed that the relative qPCR expression levels were consistent with the trend of data obtained by transcriptome analysis, indicating that the transcriptome data were reliable.

### Transcription factor analysis of DEGs

**Fig. 6:**
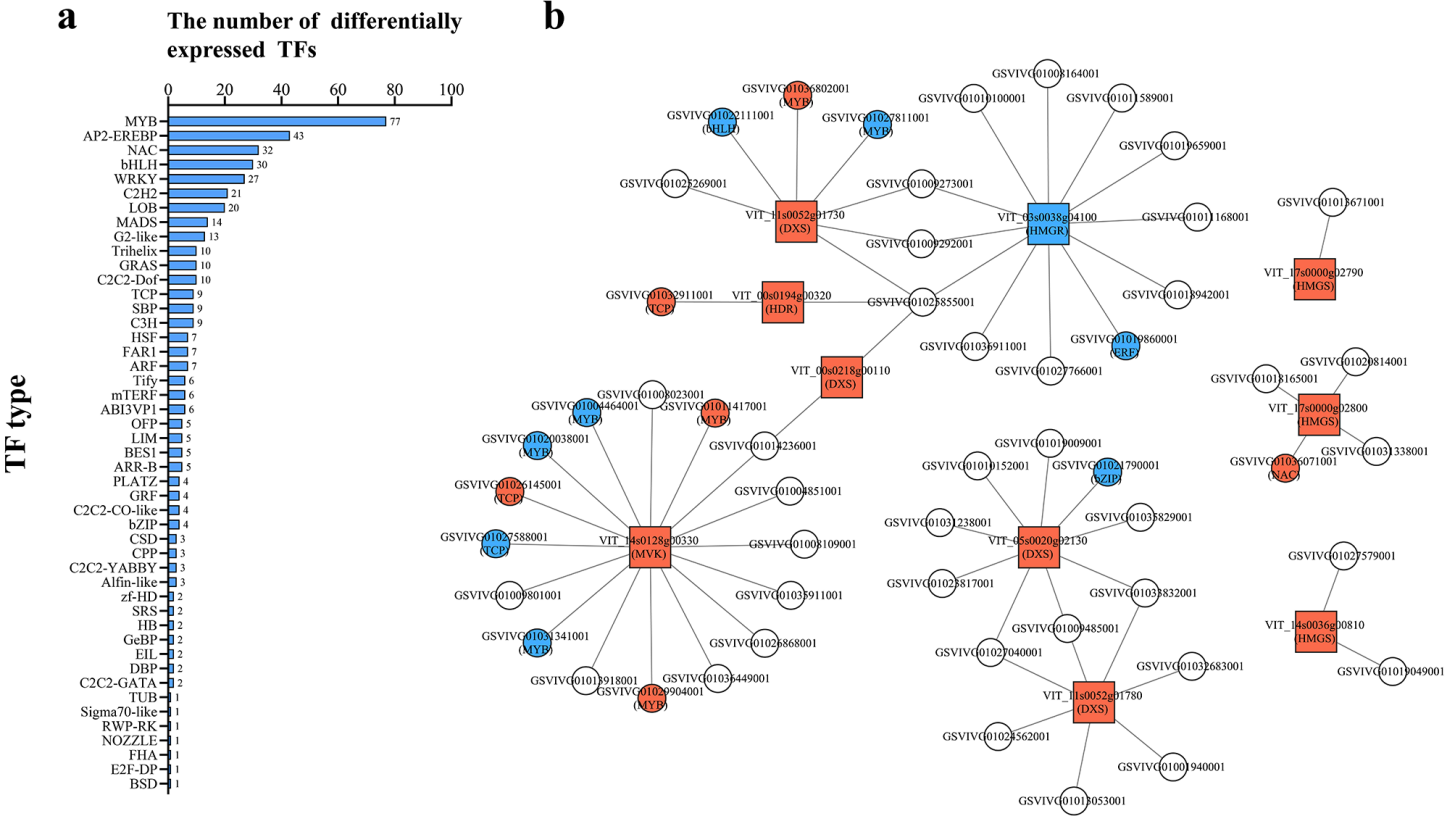
(**a**) The statistics of differentially expressed TFs. (**b**) Transcription factor regulatory networks. The circles and squares represent TFs and DEGs, respectively. Red and blue represents up- and down-regulated DEGs, respectively, in ‘Shine Muscat’ relative to ‘Heibaladuo’ table grapes

Transcription factors (TFs) are potential regulators of differential gene expression [11]. A total of 47 TF types and 442 differentially expressed TFs were found between ‘Heibaladuo’ and ‘Shine Muscat’ table grapes (Fig. 6a). The results showed that the MYB family has the biggest number of differentially expressed TFs. To determine which TFs modulate the expression of the candidate DEGs identified in this study, the potential regulatory interactions between TFs and DEGs were extracted from PlantRegMap database. A transcriptional regulatory network, involving 53 TFs and 10 target genes, is presented in Fig. 6b, and specific information is provided in Table S5. The results showed that GSVIVG01025855001 (belonging to the *C2H2* gene family) was able to regulate most of the candidate DEGs; it is likely that this TF is key to the regulation of monoterpene biosynthesis. By comparing the transcript abundance levels of TFs and candidate genes between ‘Heibaladuo’ and ‘Shine Muscat’, we found that VIT_11s0052g01730 (*VvDXS*) expression was positively regulated by GSVIVG01036802001 (*MYB*) and negatively regulated by GSVIVG01022111001 (*bHLH*) and GSVIVG01027811001 (*MYB*). VIT_14s0128g00330 (*VvMVK*) was modulated by the largest number of TFs, being positively regulated by three TFs (GSVIVG01011417001, *MYB*; GSVIVG01026145001, *TCP*; and GSVIVG01029904001, *MYB*) and negatively regulated by four TFs (GSVIVG01004464001, *MYB*; GSVIVG01020038001, *MYB*; GSVIVG01027588001, *TCP*; and GSVIVG01031341001, *MYB*). VIT_17s0000g02800 (*VvHMGS*), VIT_03s0038g04100 (*VvHMGR*) and VIT_00s0194g00320 (*VvHDR*) were positively regulated by GSVIVG01036071001 (*NAC*), GSVIVG01019860001 (*ERF*) and GSVIVG01032911001 (*TCP*), respectively, whereas GSVIVG01021790001 (*bZIP*) could negatively regulate VIT_05s0020g02130 (*VvDXS*) expression. In addition, the TFs that regulated VIT_14s0036g00810 (*VvHMGS*), VIT_17s0000g02790 (*VvHMGS*), VIT_11s0052g01780 (*VvDXS*) and VIT_00s0218g00110 (*VvDXS*) did not display differential expression between the two grape cultivars.

### Cloning and bioinformatics analysis of *VvIDI*

Based on the isoprenoid biosynthesis pathway found in plants, the molar ratio of IPP to DMAPP needed to make various terpenoid classes has already been calculated. For monoterpenes, the ratio of IPP and DMAPP was 1:1; for sesquiterpenes, the ratio was 2:1; and for diterpenes and carotenoids, the ratio was 3:1 [12, 13]. Thus, by controlling the conversion of IPP to DMAPP, *IDI* may play an important role in regulating the biosynthesis of the major types of monoterpene products formed. So, we performed some bioinformatics analyses to systematically analyze the VvIDI protein.

**Fig. 7:**
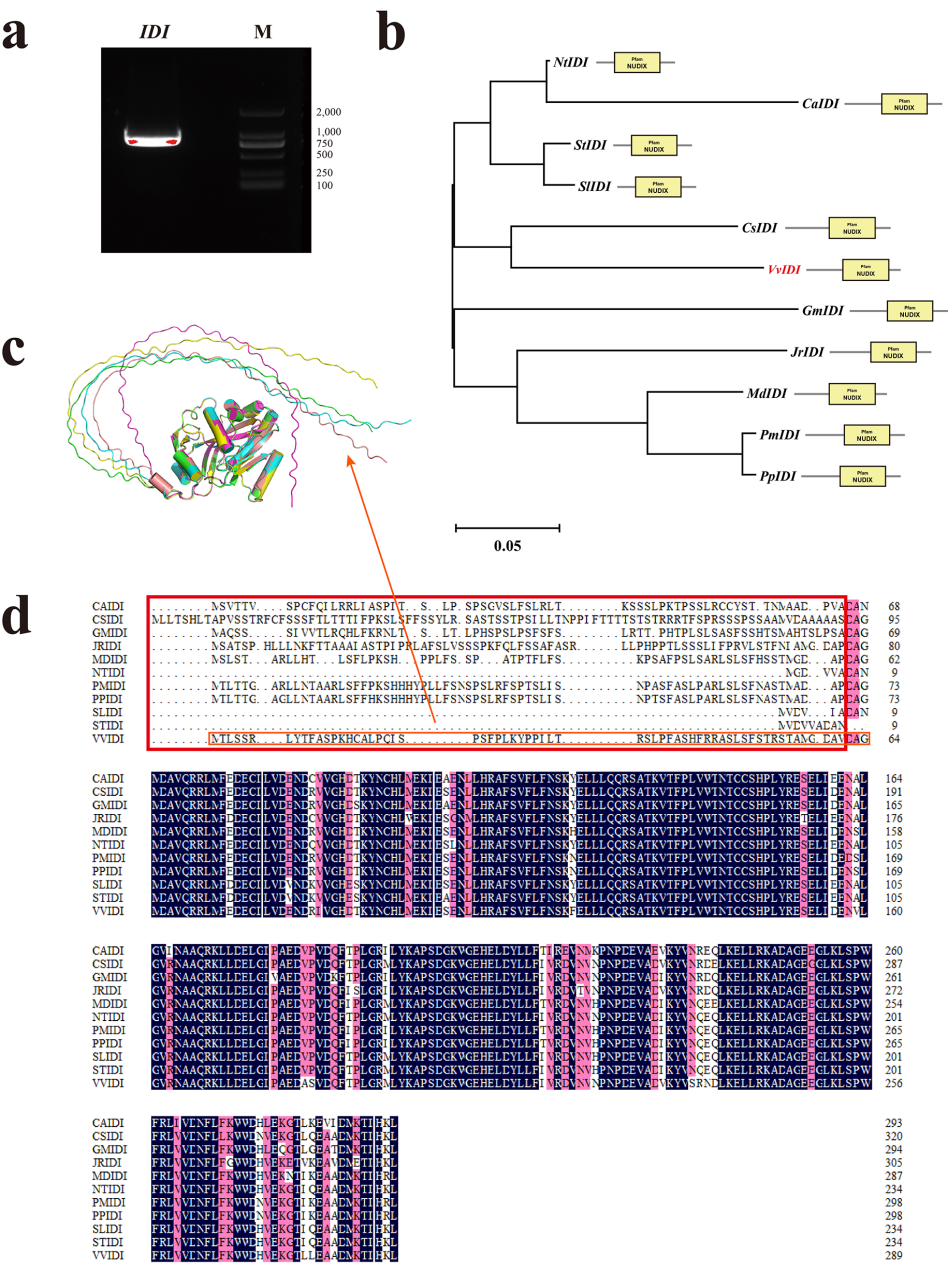
(**a**) Amplification of *VvIDI* from ‘Shine Muscat’ table grape. M represents DNA maker. (**b**) Phylogenetic and conserved domain analysis of IDI proteins from different species. The IDI protein sequences were obtained from *Nicotiana tabacum* (Nt), *Capsicum annuum* (Ca), *Solanum tuberosum* (St), *Solanum lycopersicum* (Sl), *Camellia sinensis* (Cs), *Vitis vinifera* L. (Vv), *Glycine max* (Gm), *Juglans regia* (Jr), *Malus domestica* (Md), *Prunus mume* (Pm) and *Prunus persica* (Pp). VvIDI is highlighted in the phylogenetic tree as a red font. ClustalW was used to align the pep sequences of each IDI protein, and the neighbor-joining method was used to construct the phylogenetic tree with the following settings: bootstrap method for phylogeny test; bootstrap replication was set to 1,000; *p*-distance method was set for substitution model. The branch lengths represent evolutionary distances. (**c**) The protein tertiary structure analysis of VvIDI. The five protein tertiary structures of VvIDI were predicted using AlphaFold2. Different structures are annotated in different colors. (**d**) The multiple sequence comparison of IDI proteins from different species. Amino acids with 100% homology are highlighted in black. Amino acids with homology greater than 75% are highlighted in red

To obtain the full-length coding sequence (CDS) of *VvIDI*, the fragment of *VvIDI* was amplified using the specific primers. The gel electrophoresis result is shown in Fig. 7a and Figure S1. To comprehensively analyze the phylogenetic relationship among and the interspecific differences of IDI protein, the phylogenetic tree and multiple sequence alignment with VvIDI sequence, compared with its homologs from other species, were performed. The result showed that VvIDI protein shares a close relationship with *Camellia sinensis* (CsIDI), both containing a NUDIX conserved domain that has been reported as a metal-binding and catalytic site (Fig. 7b). According to the sequence results, the protein tertiary structure prediction was performed using AlphaFold2 and shown in Fig. 7c. The prediction results indicate that the VvIDI protein has a long extension at the N-terminal, consisting of 64 amino acids. This extension is characterized as a disordered region by PONDR software (Figure S2). Multiple sequence alignment analysis showed that the sequence differences of each IDI proteins were concentrated at N-terminus (Fig. 7d). The encoded proteins in NtIDI, SlIDI and StIDI showed a shorter N-terminal extension than the IDI proteins of other species.

### A 64-amino acid extension sequence at the N-terminus can guide the VvIDI protein to target the chloroplast

**Fig. 8:**
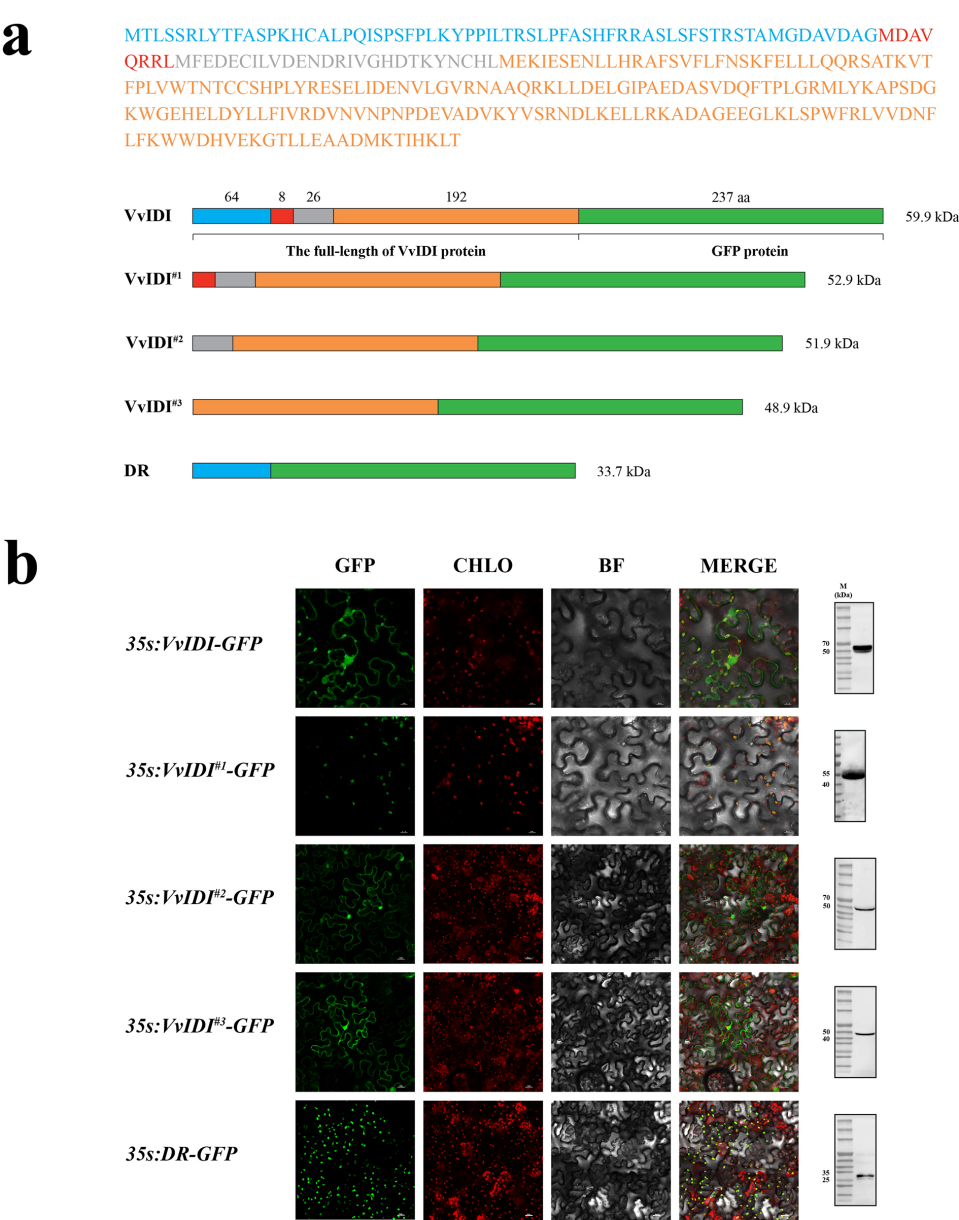
(**a**) Full-length and truncated forms of VvIDI linked to green fluorescence protein. VvIDI, the full-length VvIDI protein; VvIDI^#1^, a truncated VvIDI protein lacking the 64-amino acid extension at the N-terminus; VvIDI^#2^, a truncated VvIDI protein lacking the N-terminal 72-amino acid sequence; VvIDI^#3^, a truncated VvIDI protein lacking the N-terminal 98-amino acid sequence; DR, the N-terminal 64-amino acid sequence (the disordered region). (**b**) Subcellular localization and western blotting analysis of VvIDI. CHLO represents chloroplast; BF represents bright field; and M represents protein maker

In our previous analysis, we had found that VvIDI protein has a disordered extension, containing 64 amino acids at the N-terminus, and speculated that this extension sequence may paly important role in protein targeting. To explore the influence of this extension in VvIDI protein targeting, we performed TargetP-2.0 software to predict the subcellular location of the VvIDI protein. The predicted results showed that VvIDI protein is located in chloroplast and contain a chloroplast transfer peptide (cTP) before the 64th amino acid at the N-terminus (Figure S3). To verify the accuracy of these predicted results, the full-length coding sequence of *VvIDI* and the truncated *VvIDI* sequence lacking the sequence encoding 64, 72 and 98 amino acids at the N-terminus (*VvIDI*^*#1–3*^) were respectively inserted into the overexpression vector (pGWB505) and fused to the N-terminus of EGFP (Fig. 8a). Confocal microscopy associated with image overlay techniques demonstrated that, the fluorescence signals from the *35S: VvIDI*- and *35S: VvIDI*^*#1*^-*EGFP* fusion protein coincided with chromoplast autofluorescence, whereas the most of the fluorescence signals from *35S: VvIDI*^*#2*^- and *35S: VvIDI*^*#3*^-*EGFP* fusion proteins were observed in the nucleus and cytoplasm (Fig. 8b), which indicated that the chloroplast transfer peptide is located before the 72nd amino acid. Confusingly, combined with the subcellular localization result of VvIDI^#1^, the chloroplast localization of VvIDI remained unchanged despite the absence of the 64 amino acids at the N-terminus, which contradicted the previous predicted results. In plants, the average length of a plastid transit peptide is 50 amino acids [14], which means that the 64-amino acid at the N-terminus should contain all or part of the cTP. Therefore, the disordered region sequence encoding 64 amino acids was cloned into the same overexpression vector (*35S: DR-EGFP*). The result showed that the 64 amino acids can guide the EGFP protein to target the chloroplast (Fig. 8b), which reversely confirmed the chloroplast targeting function of the 64-amino acid extension sequence at the N-terminus of VvIDI protein. The result of western blotting showed that the electrophoresis bands were consistent with the protein size of VvIDI-, VvIDI^#1–3^- and DR-GFP fusion protein, which further validated the observation that the fluorescence signals were from these fusion proteins (Fig. 8b). The full uncropped gels and blots are shown in Figure S4.

### Overexpression of IDI inhibits monoterpene biosynthesis

**Fig. 9:**
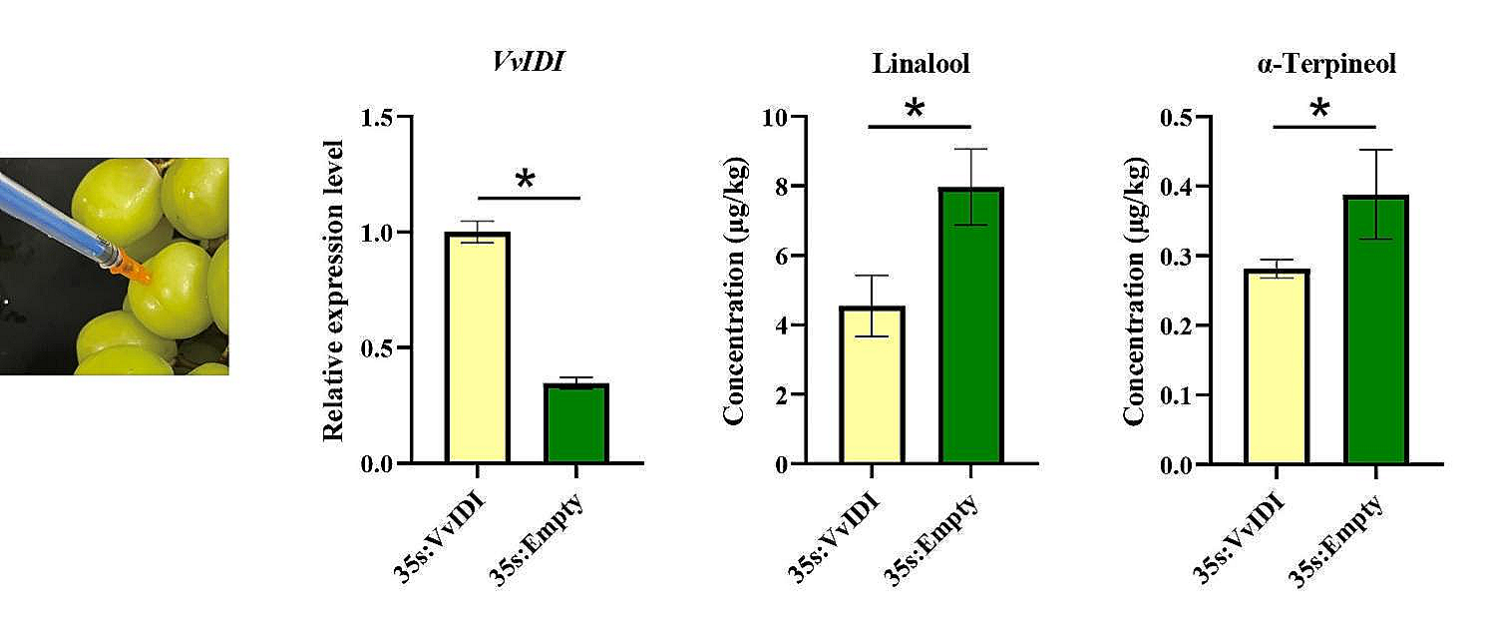
Transient expression analysis of *VvIDI* in ‘Shine Muscat’ grape berries. Error bars represent the standard deviation, SD (*n* = 3), and an asterisk (*) above the bars represents a significant difference (*p* < 0.05)

As mentioned earlier, there were particularly large fold-change differences in expression of the gene *VvIDI* between the two cultivars, and we speculated that *VvIDI* may negatively regulate monoterpene biosynthesis. To validate the function of *VvIDI* in the synthesis of monoterpenes, *Agrobacterium tumefaciens* containing pGWB505-*VvIDI* or pGWB505-*EMPTY* were infiltrated into berries of the high-monoterpene cultivar ‘Shine Muscat’ grape berries. Transient expression results showed that the expression levels of *VvIDI* were significantly increased in *VvIDI-*overexpression grapes compared to empty vector control grapes, which indicated that the overexpression vector induced the high expression of *VvIDI*. GC-MS analysis showed that the concentrations of linalool and α-terpineol were lower in pGWB505-*VvIDI* grape berries than in pGWB505-*EMPTY*, indicating that *VvIDI* can inhibit the biosynthesis of monoterpenes (Fig. 9).

## Discussion

Monoterpenes represent a group of chemicals widely distributed throughout plants, the basic structure of which consists of two linked isoprene units (C_10_H_16_) [15], which primarily contribute to the organoleptic properties observed in many fruits. This feature is particularly apparent in grapes. Most of the monoterpenes in grapes have extremely low odor detection thresholds and represent important components of the aromas of grapes, imparting sweet, floral and alcoholic notes to the berries (and to wine) [16]. With widespread applications of genomics, transcriptomics, proteomics and metabolomics in the post-genome era, multi-omics data integrative analysis has become an indispensable strategy for uncovering the biological mechanisms underlying complex traits. To obtain a better and deeper insight into the formation of monoterpenes, we tried to identify the candidate limiting genes by transcriptomics and GC-MS. By comparing differences in gene expression with those in monoterpene concentration, the results indicated that monoterpenes may be positively correlated by five *VvDXS*s, one *VvHDR*, three *VvHMGS*s and one *VvMVK* gene, while one *VvIDI* gene and one *VvHMGR* gene may negatively correlate monoterpene biosynthesis (Fig. 4). Previous studies have reported that terpene accumulation in grape and other plants was correlated with gene expression in the MEP pathway [17], such as *VvDXS*, *VvDXR*, *VvHDR* and *VvGPPS* [18–20]. Of the corresponding proteins, DXS is the first enzyme involved in the MEP pathway [21]. Battilana et al. [18] and Emanuelli et al. [22] reported the co-localization of *VvDXS* with a major quantitative trait locus (QTL) for monoterpene concentration positioned on chromosome 5 of grapevine. In addition, another study found that combining overexpression of both *VvTPS56* and *VvDXS1* could further increase monoterpene production in ‘Shine Muscat’ table grapes [23]. In our study, all *VvDXS* genes showed greater expression in the high-monoterpene ‘Shine Muscat’, a Muscat-type table grape, a finding which is in agreement with the earlier studies described above that confirmed the important role of *VvDXS* in determining Muscat-type flavor in grape. With regard to *HMGR*, an earlier study found that *VvHMGR1* exhibited a relatively high expression level during fruit development of ten grape cultivars, especially in cultivars with yellow rose fragrance [24]. However, in our study, we found that the expression of *VvHMGR1* in ‘Heibaladuo’ (a non-rose fragrance cultivar) was higher than in ‘Shine Muscat’ (a rose fragrance cultivar), a finding which was opposite to the conclusion of the previous study. We speculate that the sample size of the previous study was too small to reflect the correct expression pattern of *VvHMGR1* in various grape cultivars.

Plant growth and development depend heavily on TFs to control gene expression [25]. However, few reports have been published on the direct transcriptional regulation of terpene synthase (*TPS*) genes or genes controlling the upstream substrate biosynthesis pathway in grape. In previous studies, the TF gene *MYB24*, as a modulator of light responses, was shown to bind to the promoter region of *TPS35*, which was broadly triggered to effect terpene biosynthesis during grape berry ripening [26]. *VviWRKY40* is a TF which binds to the *VviGT14* promoter, negatively regulating the expression of *VviGT14* and further influencing the inter-conversion of free and glycosylated monoterpenes in ripening grape berries [17]. Another study found that the expression of three TF genes involved in the terpenoid backbone biosynthesis pathway, namely *VviERF3L*, *VviGATA5L* and *VviGT-2 L*, positively co-regulated expression of *VviHDR* and *VviGT14*, with significant correlation coefficients between expression levels of TFs and terpenoid biosynthesis genes of greater than 0.78 [27]. In our study, we performed network analysis of the correlations between expression levels of various TFs and the candidate DEGs (Fig. 6). The results showed that the candidate DEGs may be regulated by various TFs, including *MYB*, *bHLH*, *TCP* and so on. By comparing the expression level of the above TFs between ‘Heibaladuo’ and ‘Shine Muscat’ table grapes, we found that, of the differentially expressed TFs, the RNA transcripts belonging to the *MYB* gene family were the most abundant, indicating that the *MYB* gene family may play an important role in gene expression related to monoterpene biosynthesis. These results need to be further confirmed by using DNA pull-down, yeast one-hybrid and dual-luciferase assays.

Isopentenyl diphosphate isomerase (IDI), which plays an important role in maintaining a dynamic optimal balance between concentrations of IPP and DMAPP, has been widely investigated in the fields of the food industry, active plant biochemical extraction and fruit quality. *IDI*s have been identified and can be generally divided into two subtypes in many species, such as *Gossypium barbadense* [28], *Arabidopsis thaliana* [12] and tobacco [29]. Previous studies have revealed that cytosol-targeted IDI1 protein in *Artemisia annua* and plastid-targeted IDI1 protein in tomato can facilitate artemisinin and carotenoid biosynthesis, respectively [13, 30]. In Arabidopsis, *atidi1/atidi2* double mutants exhibited dwarfism and male sterility, which reflect their important function in plant growth and development [31]. However, little is known about their function in the synthesis of monoterpenes. In our study, the expression level of VIT_04s0023g00600 (*VvIDI*) in ‘Heibaladuo’ table grapes was higher than that in the high-monoterpene ‘Shine Muscat’ cultivar (Fig. 4), which suggests that *VvIDI* may negatively regulate the synthesis of monoterpenes. As a consequence, a recombinant overexpression plasmid containing *VvIDI* was constructed and infiltrated into the grape berries of ‘Shine Muscat’ to assess its biological functions in monoterpene biosynthesis. The results showed that overexpression of *VvIDI* decreased the accumulation of linalool and α-terpineol in high-monoterpene grape berries compared with the empty vector (Fig. 9). We speculate that the overexpression of *VvIDI* was able to control the ratio of IPP and DMAPP and shift the reaction from monoterpene (IPP: DMAPP = 1:1) toward sesquiterpene (2:1) and diterpenoid (3:1) biosynthesis pathways, causing decreased synthesis of monoterpenes as a consequence of substrate (GPP) limitations.

Disordered regions play a pivotal role for cellular processes ranging from transcriptional control and cell signaling to subcellular organization [32]. In plants, the precursor proteins that are specifically targeted to the chloroplast usually contain a chloroplast transit peptide (cTP) at the N-terminus which serves as a targeting signal [33, 34]. In our study, we conducted the protein tertiary structure, disordered region, and subcellular location prediction analyses, which revealed the presence of a disordered region and cTP at the N-terminus of the VvIDI protein (Fig. 7c, Figure S2 and S3). To investigate the functional significance of this disordered region in protein targeting, the recombinant plasmids expressing EGFP-fused the full-length and truncated VvIDI were respectively agroinfiltrated into tobacco leaves. The subcellular location results showed that VvIDI was targeted to the chloroplast, and the N-terminal 64-amino acid sequence contains a cTP capable of guiding the VvIDI protein to target the chloroplast, which suggest the N-terminal disordered region plays a crucial role in VvIDI protein targeting. Regarding the reason why the *35S: VvIDI*^*#1*^*-EGFP* fusion protein was targeted to the chloroplast, we speculated that the *35S: VvIDI*^*#1*^-*EGFP* fusion protein may interact with a certain chloroplast protein, resulting in its chloroplast localization.

## Conclusions

In this study, the candidate genes and transcription factors correlated with monoterpenes were identified, based on RNA-Seq and GC-MS analysis. Among the candidate genes identified, *VvIDI* was confirmed to negatively regulate monoterpene accumulation through a transient gene expression experiment. Meanwhile, a disordered extension containing 64 amino acids at the N-terminus was confirmed its function in guiding the VvIDI protein to target the chloroplast. In summary, this present study provided novel detailed information and insights into monoterpene biosynthesis, with the ultimate goal of identifying the mechanisms behind improving the flavor quality of grape products, both table grapes and grapes for winemaking.

## Methods

### Plant materials

Berries of ‘Heibaladuo’ and ‘Shine Muscat’ table grapes were collected on 13 July, 2021 and 15 August, 2021, respectively, from the Grape Germplasm Resources Base at Cixi, Zhejiang Province, China (30°15′18.28″ N, 121°21′58.42″ E). The climate, and physical and chemical properties of the soils in this area have been described in an earlier study [35]. All the samples were snap-frozen with liquid nitrogen and stored at − 80 °C prior to GC-MS analysis or RNA isolation.

### Measurement of general properties

A digital refractometer (ATAGO, Guangzhou, China) was used to determine the total soluble solids (TSS; Brixo) for each berry sample. A neutralization titration assay was used to determine the titratable acidity (TA; mmol/100 g). An electronic balance was used to determine the berry fresh weight (FW; g) of 20 berries.

### Extraction and GC-MS analysis of aroma compounds

A sample (50 g) of berries from each of the two grape cultivars being studied (‘Heibaladuo’ and ‘Shine Muscat’) was carefully destemmed, deseeded and ground. After centrifuging at 10,000 rpm for 30 min at 4 °C, the clear juice supernatant (6 mL) was transferred into a 20 mL glass vial (Agilent Technologies, Santa Clara, CA, USA) containing 2.0 g NaCl. An aliquot (4 µL) of internal standard (2-Octanol, 53.84 mg/L) was added into the pulp juice to quantify each aroma compound. The subsequent experimental manipulations, including the device model, instrument parameters, temperature program and volatile aroma compound quantification, were performed as described in [35].

### RNA isolation, sequencing and transcriptome data analysis

Total RNA of the two berry samples (‘Heibaladuo’ and ‘Shine Muscat’) was isolated using a Plant Total RNA Isolation Kit (Vazyme, Nanjing, China). Then, an Agilent 2100 bioanalyzer (Thermo Fisher Scientific, Waltham, MA, USA), a Nanodrop One spectrophotometer (Thermo Fisher Scientific), and gel electrophoresis were used to evaluate the quantity and quality of total RNA. The RNA-Seq platform was an Illumina HiSeq 2000 instrument, and the amount of RNA for sequencing of each sample was about 4.43 Gb.

After removing adapter and low-quality sequences from raw reads, the clean reads were aligned to the grape reference genome (http://plants.ensembl.org/Vitis_vinifera/Info/Index), using the program TopHat v2.0.943. The raw data were uploaded to the SRA database (Sequence Read Archive; https://www.ncbi.nlm.nih.gov/sra) under accession number PRJNA926483. During the process for detecting DEGs, the absolute values of log2(Fold-Change, Shine Muscat _FPKM_/Heibaladuo _FPKM_) ≥ 1 and *p* value < 0.05 were used as the thresholds with which to screen for the significant DEGs in this research. To understand the molecular functions of DEGs or the metabolic pathways and biological processes involved, GO (Gene Ontology; http://geneontology.org/) and KEGG (Kyoto Encyclopedia of Genes and Genomes; https://www.kegg.jp/) enrichment analyses were performed [36].

### RT-qPCR validation

Fifteen candidate genes, identified during the transcriptome analysis as being involved in the synthesis of monoterpenes, were selected for RT-qPCR validation. The total RNA extraction method was the same as described above. The first-strand cDNA of each sample was synthesized using First-Strand cDNA Synthesis SuperMix (Novoprotein, Shanghai, China). The expression levels of DEGs were determined using ChamQ Universal SYBR qPCR Master Mix (Vazyme). The relative expression level of DEGs was calculated using the 2^–∆∆CT^ method. Primer Premier 5 program was used to design the primers, and the sequences of each primer are listed in Table S6.

### Transcription factor analysis

The 2000-bp DNA sequences upstream of candidate genes were extracted from the grape reference genome (http://plants.ensembl.org/Vitis_vinifera/Info/Index). Then, TF-binding sites were predicted using the PlantRegMap database (http://plantregmap.gao-lab.org/regulation_prediction.php) [37].

### Cloning, protein tertiary structure, phylogenetic and multiple sequence alignment analyses of VvIDI

The full-length *VvIDI* gene was cloned using specific primers designed in Primer Premier 5 software (Table S6). The prediction of protein tertiary structure was performed using AlphaFold2 [38]. The prediction of natural disordered region was performed in PONDR software by VLS2 model (http://pondr.com/). The neighbor-joining method (bootstrap: 1000) was used to construct the phylogenetic tree of IDI proteins in MEGA 11 software [39]. The multiple sequence alignment was displayed using DNAMAN software.

### Subcellular localization and transient overexpression analyses

The subcellular location and chloroplast transfer peptide predictions of VvIDI protein were performed using TargetP-2.0 software (https://services.healthtech.dtu.dk/services/TargetP-2.0/). The coding sequence of *VvIDI* (the full-length sequence of *VvIDI* without stop codons), *VvIDI*^*#1–3*^ (lacking the 192-, 216- and 294-bp extension sequence at the N-terminus) and *DR* (the 192-bp disordered region sequence) were individually cloned into the overexpression plasmid pGWB505 vector by the Gateway cloning technique with green fluorescent protein (GFP) sequence at the C-terminus, with its expression controlled by the CaMV 35 S promoter. The recombinant plasmid was transferred into *A. tumefaciens* strain GV3101 by electroporation. The recombinant plasmid containing Agrobacterium cultures were infected into leaves of *Nicotiana benthamiana* after the cultures were activated by the Agrobacterium infiltration solution (10 mM MgCl_2_, 10 mM MES, 200 mM Acetosyringone). Two days after agroinfiltration, leaves were sampled and VvIDI localization was observed, via GFP fluorescence, using a Leica TCS SP5 confocal laser scanning microscope (Leica Microsystems, Wetzlar, Germany).

Agrobacterium containing the recombinant plasmid or the empty vector were activated in the above infiltration solution with 0.01% Tween-20, and then used to infiltrate grape berries of ‘Shine Muscat’. For each of the three biological replicates, five infiltrated berries were screened for terpene compound composition by GC-MS at 4 days post agroinfiltration.

### Western blotting

Proteins were collected, mixed individually with SDS loading buffer and boiled for five minutes. For immunoblotting, proteins were separated by electrophoresis in 10% SDS-PAGE gels, and then transferred by blotted onto nitrocellulose (NC) membranes. The blots were probed with GFP antibodies (1:5000, TransGen Biotech, Beijing, China), followed by an HRP-conjugated secondary mouse antibody (1:5000, Abbkine Scientific, Atlanta, GA, USA). The Bio-Rad ChemiDoc Touch imaging system (Bio-Rad, Hercules, CA, USA) was used to visualize the detection signals generated by an ECL reagent (Thermo Scientific), following the manufacturer’s instructions.

### Statistical analysis

One-way analysis of variance and Student’s T-test were used to analyze the data. The histograms were drawn using GraphPad Prism 9 (Harvey Motulsky, Santiago, CA, USA). The heatmaps were drawn using TBtool v1.09876 (CJ-Chen, China) [40]. The volcano map was drawn using HIPLOT PRO (Tengyunbio, Shanghai, China) [41].

### Electronic supplementary material

Below is the link to the electronic supplementary material.

Supplementary Material 1

Supplementary Material 2

## Data Availability

The raw data were uploaded to the SRA database (Sequence Read Archive; https://www.ncbi.nlm.nih.gov/sra) under accession number PRJNA926483.
