# Supplementary material for: Transcriptomic and free monoterpene analyses of aroma reveal that isopentenyl diphosphate isomerase inhibits monoterpene biosynthesis in grape (Vitis vinifera L.)
Source: BMC Plant Biol. 2024 Jun 25;24:595. doi: 10.1186/s12870-024-05306-y (PMC11197285; doi:10.1186/s12870-024-05306-y)
Supplement: Supplementary file 2 — Supplementary Material 2 [file 12870_2024_5306_MOESM2_ESM.docx]

***Supplementary Material***

**Transcriptomic and free monoterpene analyses of aroma reveal that isopentenyl diphosphate isomerase inhibits monoterpene biosynthesis in grape (*Vitis vinifera L.*)**

**Tianchi Chen ^1,2†^, Tao Xu ^1†^, Jinnan Wang ^3†^, Tianye Zhang ^3^, Jin Yang ^3^, Lixiao Feng ^3^, Tiefeng Song ^2^, Jian Yang ^3*^ and Yueyan Wu ^1*^**

^1^ College of Biological and Environmental Sciences, Zhejiang Wanli University, Ningbo 315100, China;

^2^ College of Life Sciences, Zhejiang University, Hangzhou 310058, China;

^3^ State Key Laboratory for Quality and Safety of Agro-Products, Institute of Plant Virology, Ningbo University, Ningbo 315211, China;

^*^ Correspondence: nather2008@163.com (Jian Yang); wyy2000@zwu.edu.cn (Yueyan Wu);

^†^ These authors contributed equally to this work.

There are four Supplementary figures.

**Supplementary figures**


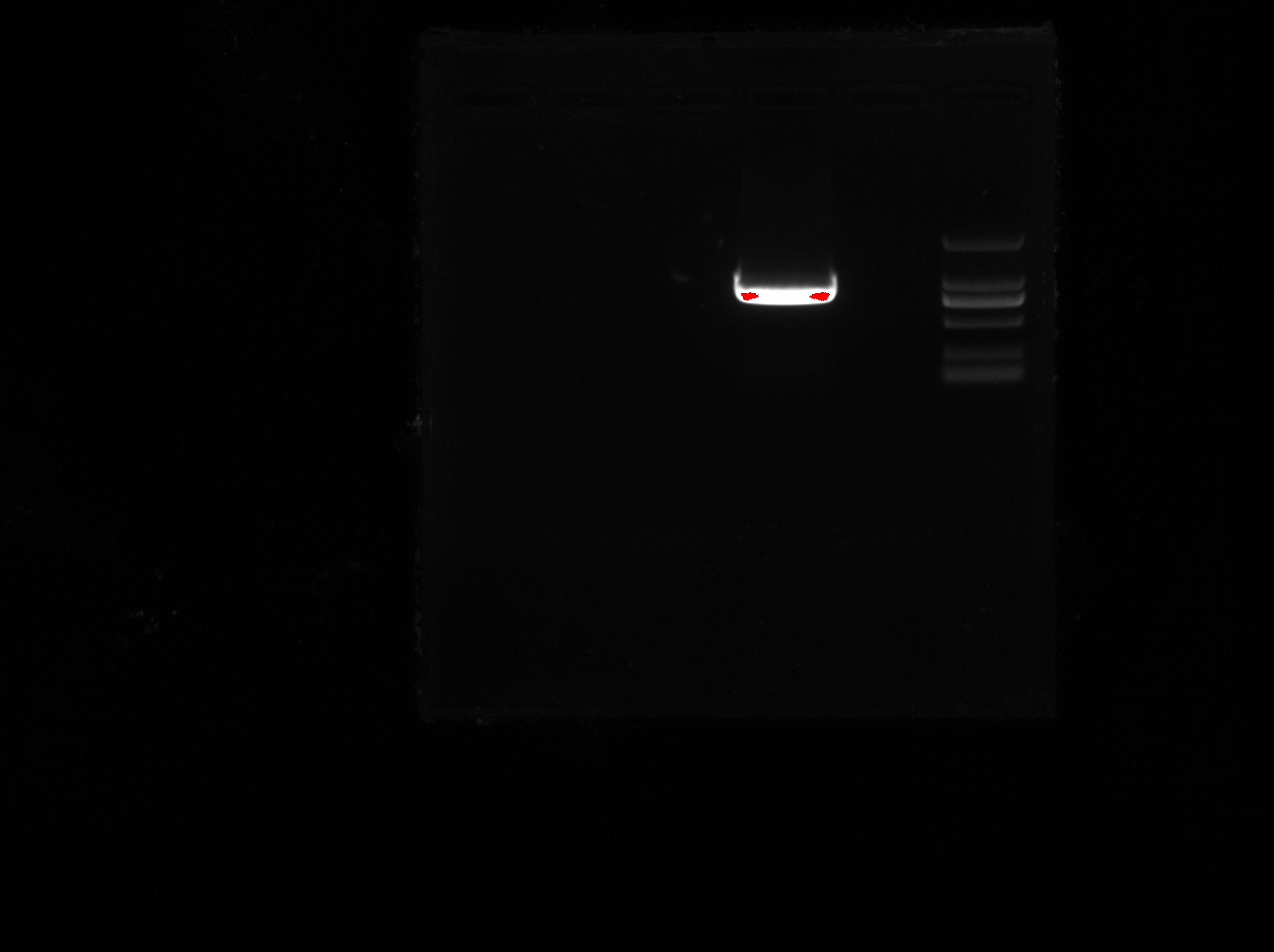


**Figure S1. The full uncropped gels electrophoresis of *VvIDI* CDS.**


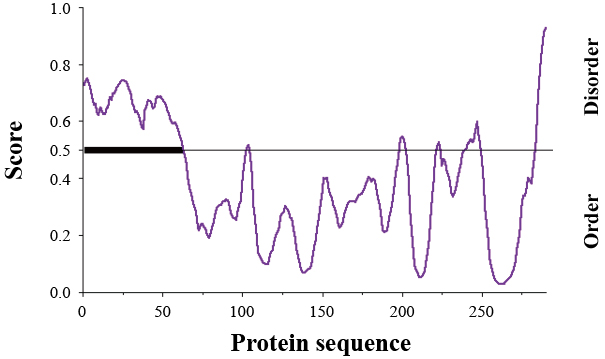


**Figure S2. Disordered region prediction of VvIDI protein.** The line above the threshold represents the disorder region.


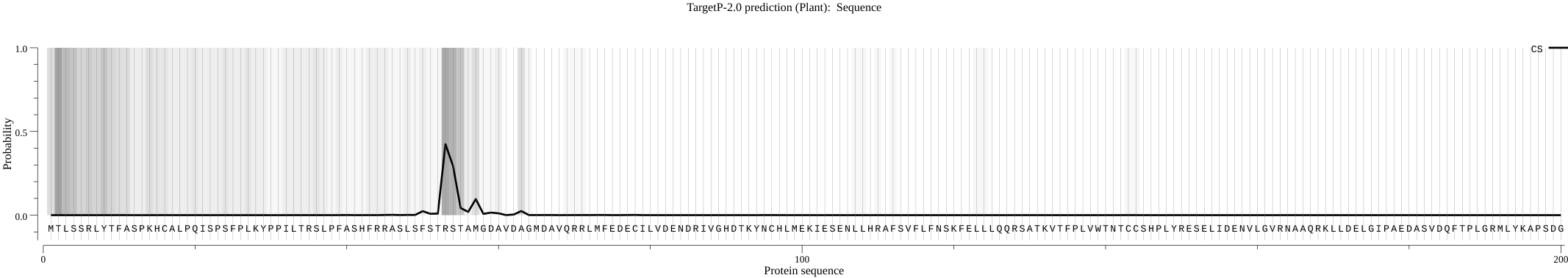


**Figure S3. The chloroplast transfer peptide prediction of VvIDI protein.**


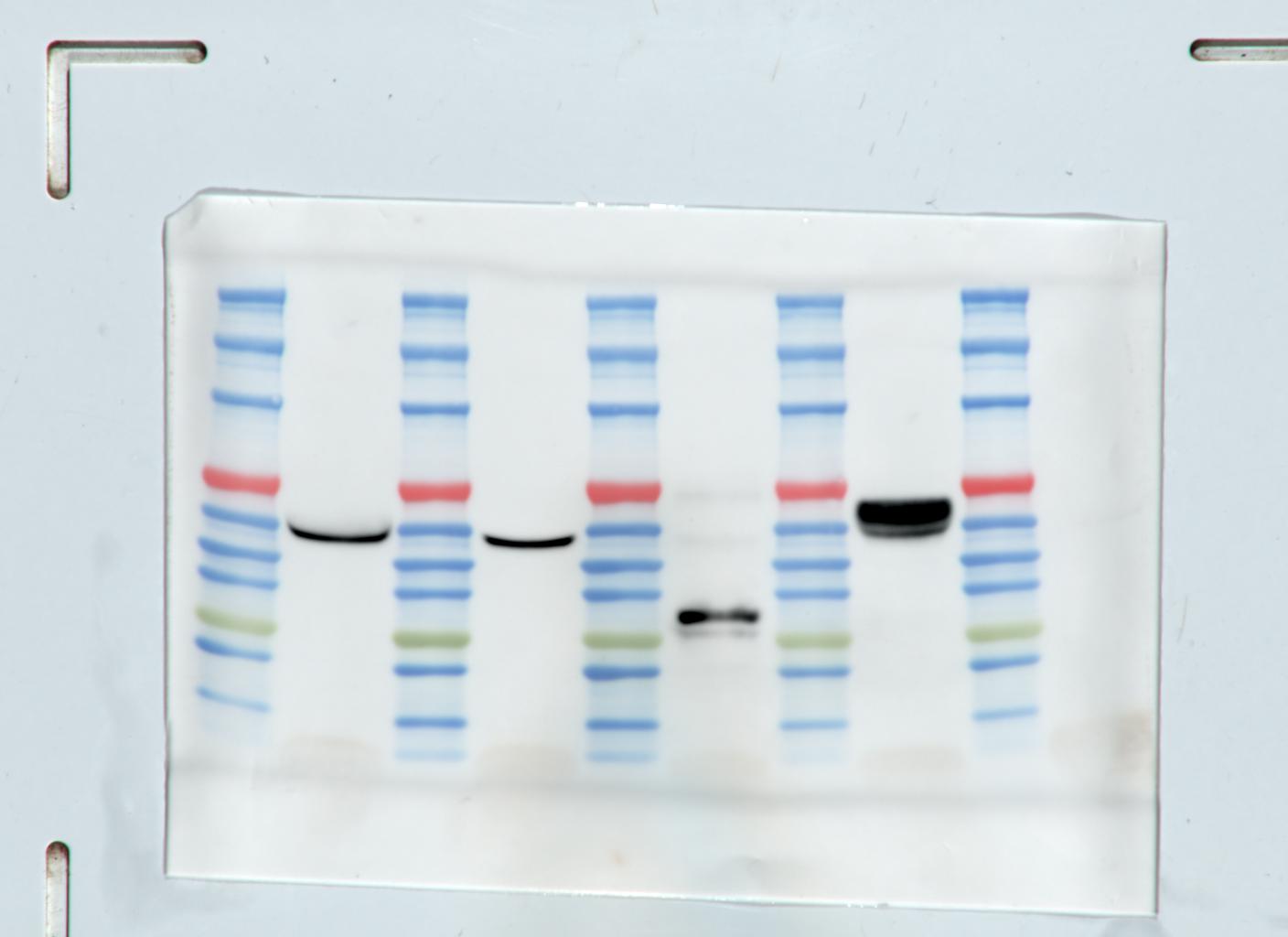

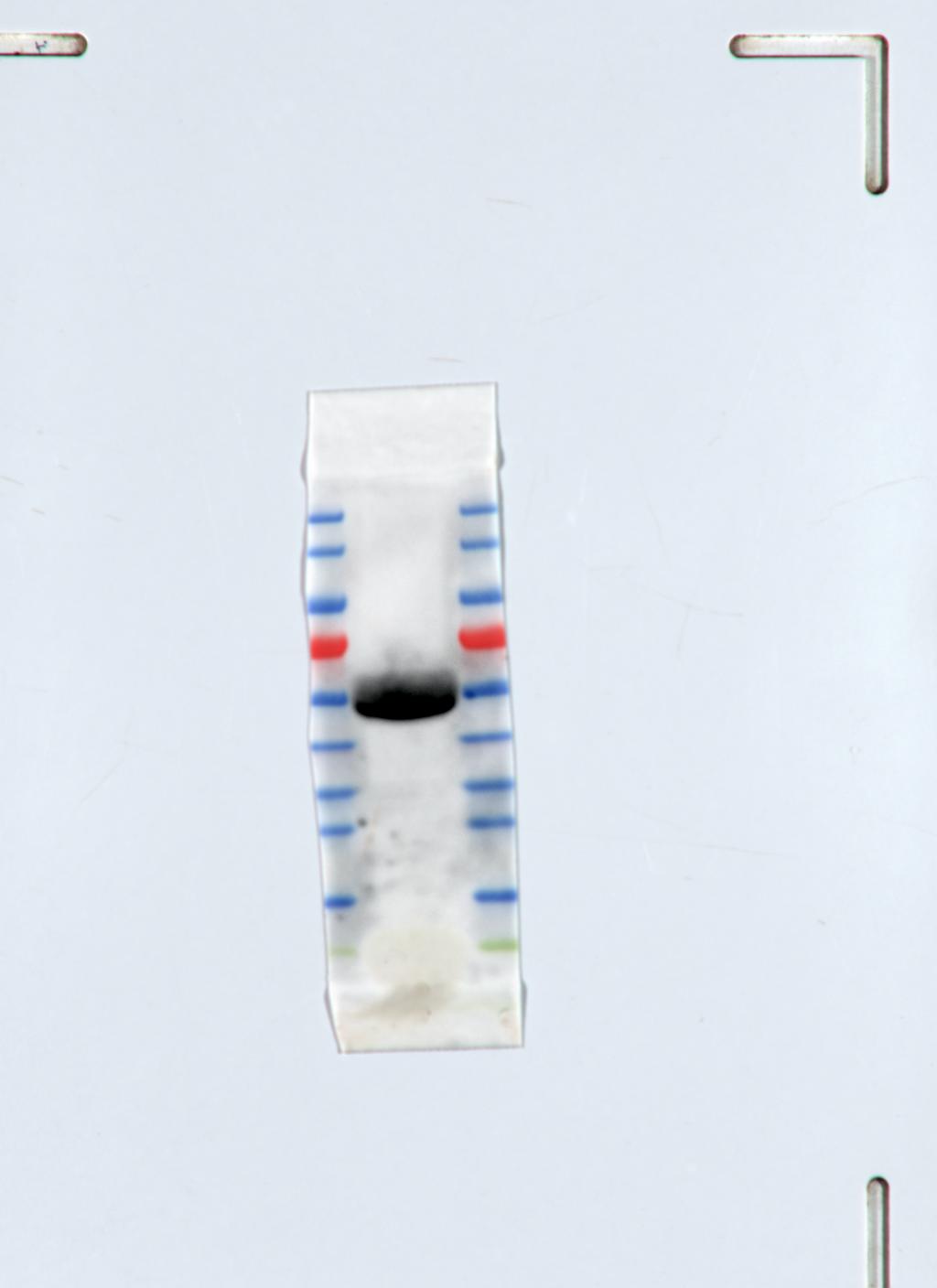


**Figure S4. The full uncropped gels and blots. From left to right:** **VvIDI^#2^-GFP, VvIDI^#3^-GFP, DR-GFP, VvIDI-GFP and VvIDI^#1^-GFP.**
